# Personalized Prediction of Postoperative Recurrence in Lung Squamous Cell Carcinoma: Integrating AI-Based Nuclear Morphometry and Clinical Data

**DOI:** 10.3390/jpm16040205

**Published:** 2026-04-06

**Authors:** Tomokazu Omori, Akira Saito, Yoshihisa Shimada, Yujin Kudo, Jun Matsubayashi, Toshitaka Nagao, Masahiko Kuroda, Norihiko Ikeda

**Affiliations:** 1 Department of Surgery, Tokyo Medical University, Tokyo 160-8402, Japan; tomokazu0720@gmail.com (T.O.); ykudo@tokyo-med.ac.jp (Y.K.); ikeda@wd5.so-net.ne.jp (N.I.); 2 Department of Anatomic Pathology, Tokyo Medical University, Tokyo 160-8402, Japan; ak-saito@tokyo-med.ac.jp (A.S.); jun-ma@tokyo-med.ac.jp (J.M.); nagao-t@tokyo-med.ac.jp (T.N.); 3 Department of AI Applied Quantitative Clinical Science, Tokyo Medical University, Tokyo 160-8402, Japan; 4 Department of Molecular Pathology, Tokyo Medical University, Tokyo 160-8402, Japan

**Keywords:** artificial intelligence, lung neoplasms, squamous cell carcinoma, prognosis, recurrence, support vector machines

## Abstract

**Background:** This study employed artificial intelligence (AI) to analyze quantitative nuclear morphological features obtained from digital pathology images to predict postoperative recurrence in patients with lung squamous cell carcinoma (LSQCC). We aimed to develop a prediction model that contributes to the realization of ‘personalized postoperative management’ tailored to individual tumor biology by integrating AI-extracted morphological features with clinical information. **Methods:** A total of 185 of the 253 surgically resected LSQCC cases were included; 136 were randomly assigned to the training set and 49 to the test set. Nuclear features from manually selected regions of interest were extracted and used to build AI-based prediction models. Three recurrence models were developed: recurrence within 2 years, within 5 years, and a three-category model (≤2 years, 3–5 years, >5 years or no recurrence). Support vector machine (SVM) and random forest (RF) algorithms were applied to each, yielding six predictive models. An ensemble approach was used to calculate AI-based risk scores, and a “total risk score” was developed by integrating these with the pathologic stage. **Results**: All six AI models demonstrated stable predictive performance, with AUC values ranging from 0.76 to 0.91. Kaplan–Meier analysis showed that the total risk score provided the most precise risk stratification (*p* < 0.005), with clearer separation between risk groups than the AI-based risk score alone. **Conclusions:** The integration of AI-based nuclear morphology analysis and clinical data provides an objective and practical tool for personalized postoperative management in LSQCC. This approach enables tailored clinical decision-making by identifying patients at high risk for early recurrence and customizing postoperative treatment plans to meet the specific needs of each individual.

## 1. Introduction

Non-small cell lung cancer (NSCLC) constitutes the majority of all lung cancer cases, with the squamous cell subtype accounting for 20–30% of cases [1,2]. Despite advances in cancer treatment, including molecular targeted therapies and immune checkpoint inhibitors, therapeutic options specific to LSQCC remain limited [3,4,5]. Consequently, patients with advanced LSQCC and postoperative recurrence often face significant treatment challenges. Moreover, early-stage LSQCC has been reported to have a poorer prognosis than early-stage lung adenocarcinoma (LUAD), highlighting the clinical importance of early postoperative recurrence [6,7,8].

Early recurrence (ER), defined as recurrence within 2 years after pulmonary resection, occurs in approximately 50% of completely resected stage I lung cancer [9,10,11]. These findings underscore the need to identify factors associated with postoperative recurrence, particularly ER, in resected LSQCC. However, current postoperative management relies primarily on pathologic stage, which may not fully capture the biological heterogeneity of each tumor. As personalized medicine continues to advance, there is an urgent need for more precise tools that facilitate individualized risk assessment.

Recent advances in artificial intelligence (AI) have significantly transformed digital pathology. Whole-slide imaging (WSI) combined with machine learning has improved diagnostic accuracy, reproducibility, and workflow efficiency [12,13,14,15]. In lung cancer, AI-based approaches have also been applied to tumor classification and prognostic assessment using quantitative nuclear morphological features [16,17]. In addition to these developments, our research group has established a unique AI-based morphometric method and demonstrated its strong predictive performance across multiple malignancies, including renal cell carcinoma, bladder cancer, and hepatocellular carcinoma [18,19,20,21].

In the present study, we applied this validated AI methodology to LSQCC to construct a predictive model based on quantitative nuclear features obtained through digital pathology. By integrating these morphometric features with clinical variables, we sought to enhance risk stratification and support personalized postoperative management. This approach may facilitate more precise clinical decision-making, such as identifying candidates who may benefit from intensified adjuvant therapy and tailored surveillance strategies according to individual recurrence risk.

## 2. Materials and Methods

### 2.1. Patients

**Figure 1:**
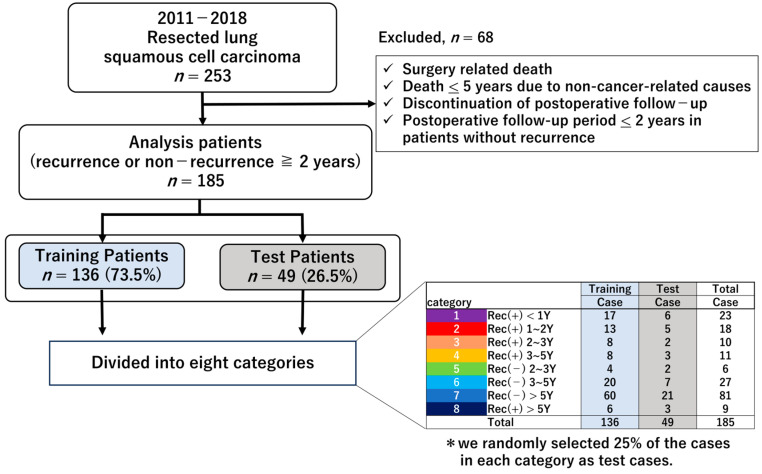
Flow diagram of patient inclusion and cohort allocation. An asterisk indicates that 25% of cases in each category were randomly assigned to the test set.

From January 2011 to December 2018, 253 consecutive patients underwent surgical resection for LSQCC at our hospital. Of these, 185 patients with postoperative recurrence or with no recurrence for more than 2 years were included. We divided 185 cases into eight categories based on recurrence and follow-up period. Approximately 25% of cases from each category were randomly assigned to a training set (*n* = 136), and the remaining 49 cases were used for testing, as shown in Figure 1. The test set was fixed for all analyses. Tumor staging was based on the 8th edition of the TNM Classification for Lung and Pleural Tumors. Clinicopathological data was obtained from medical records.

This study was approved by the Institutional Review Board of Tokyo Medical University (IRB No. T2021-0316, approved on 31 March 2022), and written informed consent was waived due to the retrospective design and anonymized data use.

### 2.2. Whole-Slide Scanning and Selection of Images

**Figure 2:**
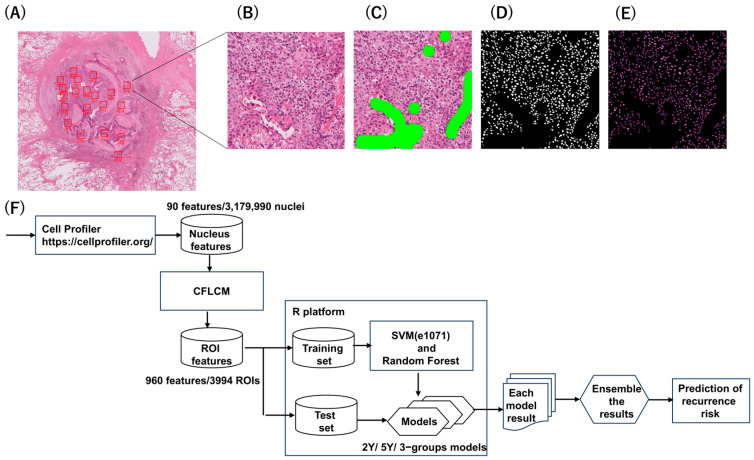
Image preparation process for the extraction of nuclei. (**A**) Twenty regions of interest (ROIs) are selected from a single slide. (**B**) Each ROI is expanded. (**C**) Fibroblasts and lymphocytes in the stroma, as well as non-tumor areas, are manually masked. (**D**) Nuclear extraction is performed, creating masked images that retain only the nuclei of cancer cells. (**E**) The masked image was overlaid on the Hematoxylin and Eosin-stained image to produce a separate nucleus image, preserving only the nuclei within the cancerous region. (**F**) Pipeline development and performance evaluation.

A WSI scanner (NanoZoomer-RS; Hamamatsu Photonics, Hamamatsu, Japan) was used to capture images of hematoxylin and eosin (HE)-stained slides of formalin-fixed, paraffin-embedded samples at a magnification of 20×. The WSI shown in Figure 2A is approximately 1 GB in size and therefore cannot be analyzed directly. Consequently, regions of interest (ROIs) were manually selected. Although automatic ROI selection would be preferable, WSI contains a variety of non-tumorous components, including normal tissue, obstructive degenerative areas, cartilage, bronchi, and blood vessels. In addition, lung squamous cell carcinoma (LSQCC) is characterized by extensive necrotic regions. Because an automated system capable of reliably extracting cancer cell regions relevant to the purpose of this study has not yet been established, manual ROI selection was performed. Furthermore, even within tumor cell nest regions, areas containing minimal infiltration of blood cells, such as lymphocytes and plasma cells, were preferentially selected. As a result, a total of 3994 ROIs (mean, 21 ROIs per slide) were extracted from WSI of 185 cases, as shown in Figure 2B. ROI size was 2048 × 2048 pixels, equivalent to 1 mm^2^.

### 2.3. Nuclear Extraction and Segmentation

ROI images obtained from the whole-slide scans included stromal fibroblasts and lymphocytes. Although the ROIs represent the tumor microenvironment, they still include microvascular endothelial cells as well as stromal components containing fibroblasts and inflammatory cells such as lymphocytes and plasma cells. In addition, artifact regions caused by surgical manipulation or slide preparation were also present. These non-tumor components were manually masked in green, and ROI images containing cancer cells only were generated. This process is illustrated in Figure 2C. Nuclear extraction was performed using the ilastik software, version 1.3.2post1 (available online: http://ilastik.org/ (accessed on 10 March 2025)), generating masked images containing only cancer cell nuclei. Some nuclei remained polymerized. For nuclear segmentation, a pix2pix-based model was used to generate a mask that excluded non-nuclear regions. The resulting mask image (Figure 2D) was overlaid onto the cancer-cell-only ROI image shown in Figure 2C, thereby producing H&E images containing nuclei only (Figure 2E).

### 2.4. Quantitative Measurement of Nuclei

The morphological and intranuclear (chromatin) textural features were quantified using CellProfiler software, version 3.1.5 (Broad Institute, Cambridge, MA, USA; available online: http://www.cellprofiler.org/ (accessed on 10 March 2025)), as shown in Figure 2F. Nucleus shape related features (e.g., size, roundness, major and minor axis length, eccentricity, and solidity, etc.) and intranuclear texture features (second angular moment, entropy, homogeneity, and using the gray-level co-occurrence matrix (GLCM) nuclear texture features total 90 features were measured for extracted total 3,179,990 nuclei. These features were input into the cell feature-level co-occurrence matrix (CFLCM), an in-house software tool registered on GitHub in March 2023 (available online: https://github.com/Shen-tokyomed/Breast_AI_CFLCM_tool/ (accessed on 10 March 2025)); this was used to calculate 960 ROI-based features such as the average, variance, and heterogeneity of the ROI [22]. The gray-level co-occurrence matrix (GLCM) has been widely used since the 1980s as a standard method for quantifying texture features in image analysis. Conventional GLCM-based co-occurrence matrices compute texture features using Haralick functions at the pixel level. In contrast, the cell feature–level co-occurrence matrix (CFLCM) replaces each individual cell nucleus with a single pixel and quantifies nuclear heterogeneity across the entire region of interest (ROI), thereby capturing spatial variations in nuclear features at the cellular level.

### 2.5. Analysis Methods

Machine learning models included SVM with linear kernels using the e1071 package, version 1.7-11, and RF using the randomForest package, version 4.7-1.1, in R, version 4.2.1 (R Foundation for Statistical Computing, Vienna, Austria; available online: https://www.r-project.org/ (accessed on 15 February 2026)). For multi-class classification, the SVM used the one-vs-one approach, and the RF used the one-vs-rest approach. Both methods used default settings in R (e1071 package). To maximize the utility of cases with follow-up shorter than 5 years, we constructed three models: (1) recurrence within 2 years, (2) recurrence within 5 years, and (3) recurrence within 2 years, between 3 and 5 years, and no recurrence over 5 years. SVM and RF were applied to each model, resulting in six models in total. The results of these six models were then combined (ensemble) to calculate an AI-based 5-year recurrence risk score.

The dataset was divided at the case level into training and test cohorts prior to model development, ensuring that ROIs from the same patient were not shared between datasets. To construct the training and test datasets, cases were stratified by time to recurrence and follow-up duration, and approximately 25% of cases from each stratum were allocated to the test dataset, while the remaining 75% were assigned to the training dataset.

Model stability within the training cohort was assessed using 5-fold cross-validation for SVM and out-of-bag error for RF. The fixed independent test cohort was not used during model training, hyperparameter tuning, or model selection, and was reserved exclusively for performance evaluation to minimize the risk of overfitting.

To develop the case-level prediction models, SVM and RF were initially trained and evaluated using individual ROI-level data. To aggregate ROI-level results at the case level, likelihood scores calculated for individual ROIs were averaged for each case, and the resulting mean value was used as the case-level likelihood score.

Model performance was evaluated using ROC curves and AUC. Kaplan–Meier analyses of recurrence-free survival were performed using Python with the lifelines package (available online: https://lifelines.readthedocs.io/, (accessed on 15 February 2026)), and other statistical analyses were conducted using IBM SPSS Statistics for Windows, version 28.0 (IBM Corp., Armonk, NY, USA). To classify the prediction results from each of the six AI models, a recurrence likelihood was obtained for each model. When both SVM and RF showed recurrence likelihoods ≥0.5, the case was defined as positive (red color); when only one of them was ≥0.5, it was defined as partial positive (orange color); and when both were <0.5, it was defined as negative (blue color). Each classification was assigned a point value (red = 2 points, orange = 1 point, blue = 0 points). The total points from all six models were summed to obtain the AI score, which was then used to classify patients into high-risk (6–10 points), middle-risk (3–5 points), and low-risk (1–2 points) groups.

**Figure 3:**
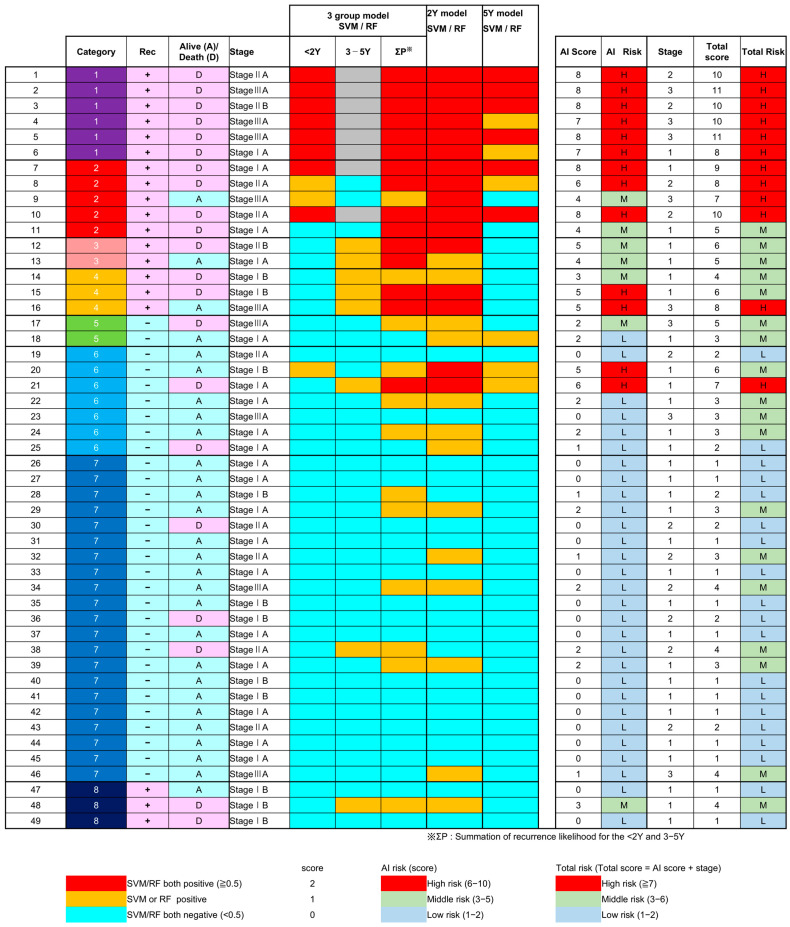
Summary of recurrence prediction results from six AI models. Each case was evaluated by six models (SVM and RF for 2-year, 5-year, and three-group model (≤2 years, 3–5 years, >5 years or no recurrence)). In the “Category” column, categories 1–8 are color-coded according to the recurrence/follow-up groups defined in Figure 1 (purple, red, pink, yellow, green, light blue, blue, and dark blue, respectively). In the “Rec” column, “+” indicates recurrence and “−” indicates no recurrence. In the “Alive (A)/Death (D)” column, A indicates alive and D indicates death, as indicated by the respective background colors. Cases were labeled as red (both positive ≥ 0.5), yellow (either positive), or blue (both negative). Scores were assigned as red = 2, yellow = 1, and blue = 0, and summed to obtain the AI score. The total score was calculated by incorporating the pathologic stage score (I = 1, II = 2, III = 3). Cases with both SVM and RF positive in the 2-year model were considered early recurrence and shown as gray, indicating no 3–5 year recurrence risk. The detailed numerical feature values for each case have been moved to Supplemental Figure S2 to enhance the readability of this overview. AI, Artificial intelligence; SVM, support vector machine; RF, random forest.

Additionally, a total score was calculated by adding the pathologic stage score (stage I = 1, stage II = 2, stage III = 3) to the AI score. We categorized patients into high-risk (≥7 points), middle-risk (3–6 points), or low-risk (1–2 points) groups according to the total score. For cases in which both SVM and RF models for the 2-year recurrence prediction were positive (≥0.5), we considered them as having a strong likelihood of early recurrence. Therefore, cases with high predicted risk of recurrence within 2 years were categorized as predominantly early-recurrence-driven and were displayed as gray areas in Figure 3. These temporal risk classifications were subsequently used for Kaplan–Meier survival analyses.

## 3. Results

### 3.1. Patient Characteristics and Quantitative Nuclear Morphological Features

Patient characteristics are summarized in Table 1. The median follow-up duration was 5.5 years. A total of 185 patients were analyzed after excluding 68 cases that did not meet the inclusion criteria. Among them, 136 patients (73.5%) were randomly assigned to the training set and 49 patients (26.5%) to the test set. No significant differences in clinicopathological characteristics were observed between the training and test sets.

ROI and nuclear extraction were manually performed for each category. In total, 3994 ROIs and 3,179,990 cancer nuclei were extracted from 185 patients, as summarized in Supplemental Table S1.

### 3.2. Predictive Performance of All Six AI Models for Postoperative Recurrence

**Figure 4:**
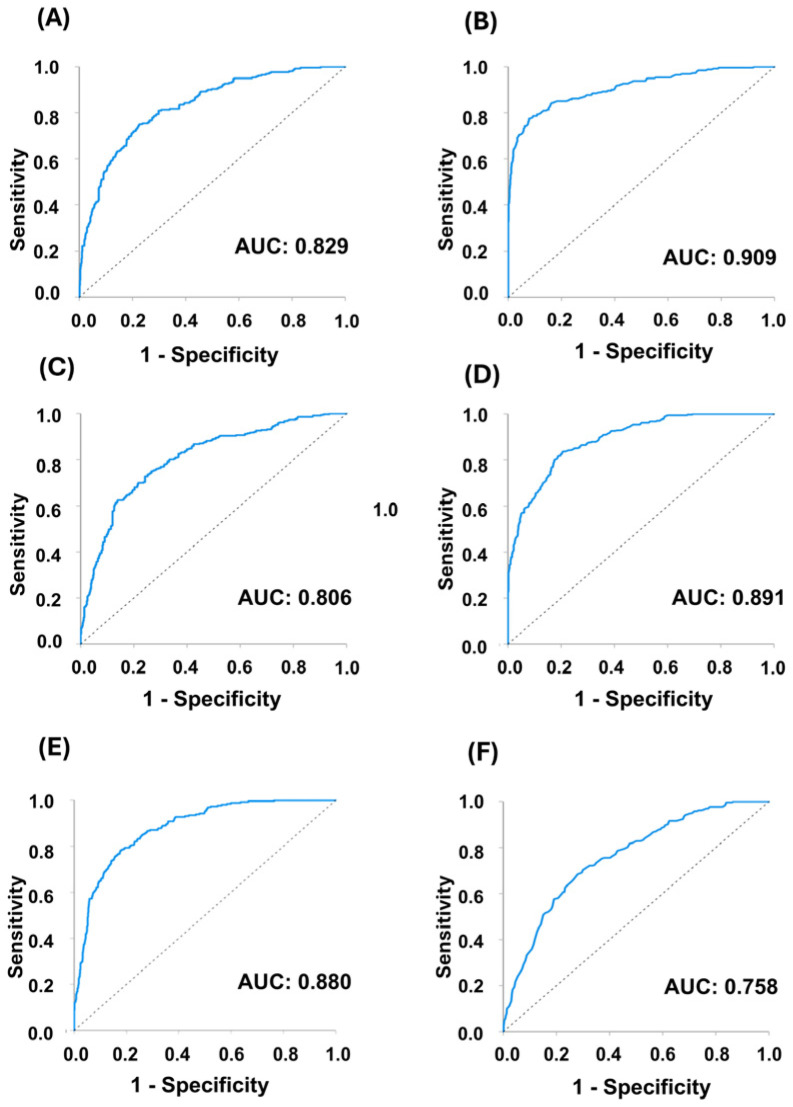
Model performance evaluation using ROC analysis. The ROC curves of recurrence within 2 years model: (**A**) SVM and (**B**) RF. The ROC curves of recurrence within 5 years model: (**C**) SVM and (**D**) RF. The ROC curves of three-category classification (≤2 years, 3–5 years, and >5 years or no recurrence) model: (**E**) SVM and (**F**) RF. The dotted diagonal line indicates chance-level performance (AUC = 0.5). ROC, receiver operating characteristic; SVM, support vector machine; RF, random forest.

**Figure 5:**
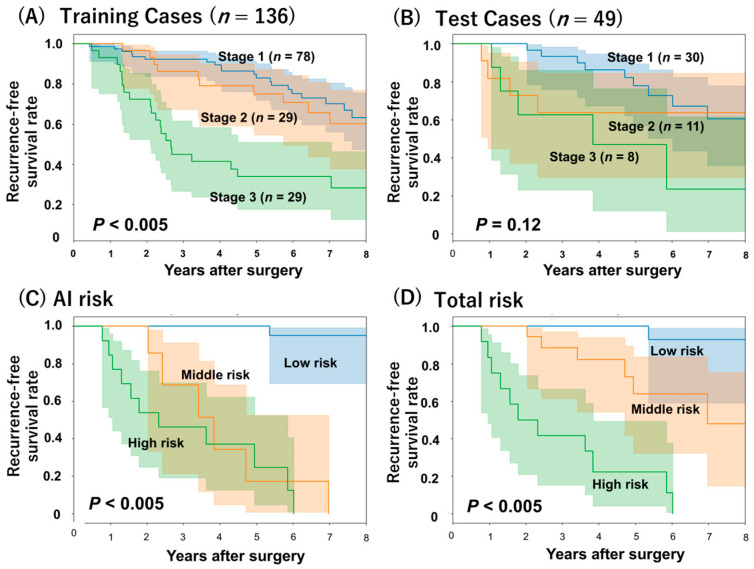
Kaplan–Meier curves for recurrence-free survival (RFS). Stage-specific RFS in (**A**) Training set, (**B**) Test set. (**C**) Risk-stratified RFS based on the AI-based risk score. (**D**) Risk-stratified RFS based on Total risk score (the AI-based risk and clinical factor). AI, Artificial intelligence.

Both SVM and RF models were trained using ROI-level data to distinguish recurrence from non-recurrence in the training set, and recurrence likelihoods were calculated for each ROI in the test set. The likelihood of each ROI was averaged on a case-by-case basis. To assess generalization performance, SVM underwent 5-fold cross-validation and RF was evaluated using out-of-bag error within the training cohort. As illustrated in Supplemental Figure S1, both approaches demonstrated stable error convergence without evidence of performance instability. All models were subsequently evaluated on the fixed independent test cohort, where consistent predictive performance further supported generalizability. The detailed classification performance and misclassification analysis for each model are summarized in the confusion matrices provided in Supplemental Tables S2–S4. The predictive results of these six models, their ROC curves, and KM survival analyses using the risk scores are summarized in Figure 3, Figure 4 and Figure 5.

Supplemental Table S5 shows the detailed breakdown of feature importance for each of the six models. In the SVM models, nuclear shape-related features accounted for a dominant proportion of the predictive power (85.8% in the 2-year model and 82.0% in the 5-year model). Among these, nuclear orientation heterogeneity was identified as the most critical factor, contributing 41.8% to the 2-year SVM model. Other influential shape-related features included the maximum radius and form factor. In contrast, Supplemental Table S5 also demonstrates that the RF models utilized a more balanced integration of features, with shape-related and intranuclear texture features contributing approximately 52% and 48%, respectively. Notably, intranuclear texture contrast—representing internal intensity variations—emerged as a significant predictive feature in the RF analysis.

### 3.3. Results of the Six AI Models

Figure 3 illustrates the predictive performance of six AI models (each three models in SVM and RF) constructed using data from the test cohort. Each model generated a recurrence probability (likelihood) for every test patient. The AI risk score, calculated by combining the results of the six models, showed good agreement with actual recurrence outcomes. Additionally, the combined score integrating pathologic stage and AI score also showed good agreement.

### 3.4. Results of ROC Curve Analysis

ROC curve analysis was performed to evaluate the predictive performance of each model. As shown in Figure 4, all six AI models showed good prediction ability, with AUC values between 0.758 and 0.909 (SVM: 0.806–0.880; RF: 0.758–0.909).

### 3.5. Survival Analysis

Kaplan–Meier survival analysis was performed to compare recurrence-free survival (RFS) among different risk groups. In the training set, as shown in Figure 5A, patients with more advanced-stage diseases had significantly shorter RFS than those with earlier-stage diseases (*p* < 0.005). In the test set, illustrated in Figure 5B, a similar trend was seen, but it was not statistically significant (*p* = 0.12). When patients were classified into low-risk, middle-risk, and high-risk groups using the AI-based risk score, presented in Figure 5C, RFS differed significantly among the three groups (*p* < 0.005). Also, the total risk score that combined AI-based and clinical information, as shown in Figure 5D also showed a significant difference (*p* < 0.005). The three survival curves showed clearer separation in the total risk model than in the AI-only model.

## 4. Discussion

In this study, we demonstrated that postoperative recurrence risk in patients with LSQCC can be effectively predicted by integrating AI-based nuclear morphology analysis with clinical information. Six AI models targeting different recurrence intervals showed stable performance, and their ensemble integration improved predictive stability and consistency. Furthermore, combining AI-based risk scores with pathologic stage enhanced risk stratification compared with either approach alone.

Accurate prediction of postoperative recurrence remains a major clinical challenge in LSQCC. Although pathologic stage remains an important component of postoperative management, it does not fully account for inter-patient biological heterogeneity, and patients with similar stages often experience markedly different clinical outcomes. Our findings indicate that quantitative nuclear morphological features extracted from routine histopathological slides provide additional prognostic information that complements conventional clinicopathological factors.

A notable advantage of the present approach is the ensemble integration of multiple models designed for different recurrence intervals, which helped mitigate model-specific bias and improved the reliability of prediction across patient subsets. The ensemble strategy enabled the integration of strengths from individual models while minimizing the impact of errors from any single model. Similar morphology-based machine-learning approaches have been reported for recurrence or prognostic prediction in other malignancies [18,19,20], and the present study extends these concepts to LSQCC, where objective postoperative risk stratification using AI-based nuclear morphology has not been well explored.

From a biological perspective, nuclear morphology reflects the downstream effects of complex molecular and cellular processes, including genomic alterations, transcriptional programs, and tumor–microenvironment interactions. In LSQCC, alterations in pathways such as TP53, NFE2L2, SOX2, and TP63 are known to influence cell-cycle regulation, differentiation, and chromatin organization, which may manifest as nuclear pleomorphism and textural heterogeneity [23,24,25,26,27,28,29]. In addition, differences in microRNA expression profiles between primary and metastatic lung squamous cell carcinoma have been reported, further supporting the concept that molecular heterogeneity underlying tumor progression may be reflected in morphological characteristics of cancer cells [30]. These identified morphometric signatures provide important biological insights. While the absolute orientation of a single nucleus is stochastic, high orientation heterogeneity at the ROI level reflects a loss of cellular polarity and disorganized tissue architecture, which are common hallmarks of high-grade malignancy in LSQCC. Similarly, increased intranuclear texture contrast suggests chromatin remodeling and genomic instability. Although these subtle intranuclear heterogeneities are often beyond the limits of human visual perception, our AI-based approach quantifies these sub-visual patterns, offering an objective assessment of tumor aggressiveness that complements traditional pathological grading. Therefore, the morphological features quantified in this study likely represent integrated tumor phenotypes relevant to recurrence risk.

This approach is readily applicable in routine clinical practice. Digital pathology is already widely used in routine practice and does not require additional tissue sampling or specialized molecular testing. By leveraging routinely available histopathological data, our model offers a practical and cost-effective tool for postoperative risk assessment. The ability to identify patients at higher risk of early recurrence may support more informed clinical decision-making, such as closer surveillance or consideration of adjuvant therapy, while avoiding unnecessary interventions in low-risk patients.

While the primary objective of this study was recurrence prediction, the present findings also suggest potential relevance to personalized postoperative management. By providing individualized risk estimates based on both morphological and clinical information, this approach may contribute to more tailored follow-up strategies. Such risk-adapted management is consistent with current concepts of precision oncology, which emphasize individualized clinical decision-making beyond genomics alone [31,32]. However, further studies are needed to determine how such risk stratification can be optimally integrated into treatment algorithms [33].

Regarding the positioning within existing prognostic frameworks, several models for lung cancer have been developed using Radiomics and genomic signatures. Genomic assays provide deep molecular insights but are often limited by high costs and the requirement for specialized infrastructure. Similarly, while Radiomics offers non-invasive macro-level analysis, it may not fully capture the cellular-level heterogeneity of the tumor. In contrast, our AI-based nuclear morphometry leverages standard HE-stained slides, offering a cost-effective and highly accessible tool for objective risk stratification. This “digital biopsy” approach provides a direct assessment of nuclear atypia, which serves as a powerful surrogate for the underlying genomic instability and tumor aggressiveness.

Regarding practical implementation, our model is designed to integrate seamlessly into the emerging digital pathology workflow. Since it relies on standard HE-stained slides, there is no requirement for additional laboratory procedures, ensuring high cost-effectiveness. In a clinical setting, this AI tool could function as a second-look system, providing pathologists with objective morphometric data to support their final prognosis. However, we emphasize that this model is currently intended as a decision-support tool to complement, rather than replace, established clinicopathological grading. Further refinement of automated ROI selection and integration with Laboratory Information Systems (LIS) will be essential for full-scale clinical adoption.

There are some limitations of this study. This was a retrospective design, and the sample size was relatively small. Large cohorts are needed to improve model accuracy and reduce overfitting. Second, although this study focused on nuclear features, incorporation of additional histological factors—such as microscopic vascular invasion, tumor necrosis, keratinization, and lymphoid infiltration—may further enhance predictive performance and clinical relevance. In addition, external validation is required to confirm the robustness and reproducibility of the model across institutions. Third, ROIs were manually selected, which may introduce selection bias. However, this manual approach was strategically employed to ensure data purity by excluding complex non-tumor components inherent in LSQCC histology—such as stroma, necrosis, cartilage, and obstructive changes—which could otherwise act as noise during AI training. Furthermore, since the prognostic significance of nuclear features was only revealed through objective AI-based quantification after ROI selection, it was not possible to bias the selection based on a priori estimation of clinical outcomes. Although these micro-ROIs were manually masked to obtain pure squamous carcinoma nuclei, as shown in Figure 2C, improving data quality, we acknowledge that this potentially introduces minor inter-observer variability. Future studies will focus on developing and validating automated ROI detection systems across multiple institutions to more accurately and efficiently isolate cancer cell nuclei. Furthermore, as LSQCC is epidemiologically more prevalent in males, our cohort was predominantly male (84.3%), which limited our ability to perform a statistically robust assessment of model fairness across genders. Although Supplemental Table S6 confirms that pathologic stage was a strong independent predictor in our multivariate analysis, we acknowledge that this study remains a single-center proof-of-concept. External validation is required to confirm the robustness of the model across different centers and more diverse populations. Despite these limitations, our findings provide a promising foundation for the development of AI-based prediction models.

## 5. Conclusions

In conclusion, the integration of AI-based nuclear morphology analysis with clinical information enables reliable prediction of postoperative recurrence in LSQCC. This approach provides an objective and practical framework for postoperative risk stratification and has the potential to support more individualized clinical management.

## Figures and Tables

**Table 1:** Patient characteristics.

Variables, *n* (%)	Overall *n* = 185	Training Patients *n* = 136 (73.5)	Test Patients *n* = 49 (26.5)	*p* Value
Sex, *n* (%)				0.823
Male	156 (84.3)	114 (83.8)	42 (85.7)	
Female	29 (15.7)	22 (16.2)	7 (14.3)	
Age, median (range)	73 (41–86)	72 (41–86)	74 (58–86)	0.258
Age category, *n* (%)				
≧65	155 (83.8)	111 (81.6)	44 (89.8)	
<65	30 (16.2)	25 (18.4)	5 (10.2)	
Smoking status, *n* (%)				0.100
Current/Former	182 (98.4)	133 (97.8)	49 (100)	
Never	3 (1.6)	3 (2.2)	0 (0)	
Clinical stage, *n* (%)				0.45
Stage I	107 (57.8)	76 (55.9)	31 (63.3)	
Stage II	45 (24.3)	33 (24.3)	12 (24.5)	
Stage III	31 (16.8)	26 (19.1)	5 (10.2)	
Stage IV	2 (1.1)	1 (0.7)	1 (2.0)	
Pathologic stage, *n* (%)				0.72
Stage I	108 (58.4)	78 (57.4)	30 (61.2)	
Stage II	40 (21.6)	29 (21.3)	11 (22.4)	
Stage III	37 (20.0)	29 (21.3)	8 (16.3)	
Pathologic N stage, *n* (%)				0.324
N0	118 (63.8)	89 (65.4)	29 (59.2)	
N1–3	56 (30.3)	41 (30.2)	15 (30.6)	
NX	11 (5.9)	6 (4.4)	5 (10.2)	
Vascular invasion, *n* (%)				1.000
Negative	66 (35.7)	49 (36.0)	17 (34.7)	
Positive	119 (64.3)	87 (64.0)	32 (65.3)	
Lymphatic invasion, *n* (%)				0.867
Negative	83 (44.9)	62 (45.6)	21 (42.9)	
Positive	102 (55.1)	74 (54.4)	28 (57.1)	
Pleural invasion, *n* (%)				0.500
Negative	113 (61.1)	81 (59.6)	32 (65.3)	
Positive	72 (38.9)	55 (40.4)	17 (34.7)	

## Data Availability

The raw data supporting the conclusions of this article will be made available by the authors upon reasonable request.

## Supplementary Materials

The following supporting information can be downloaded at: https://www.mdpi.com/article/10.3390/jpm16040205/s1, Table S1: The number of ROIs and nuclei in each category of the analyzed cases; Table S2: The results of SVM and RF models in the three-group AI model; Table S3: The results of SVM and RF models in the 2-year recurrence model. (only Test set result); Table S4: The results of SVM and RF models in the 5-year recurrence model. (only Test set result); Table S5: Relative importance of nuclear morphological and textural features in AI-based recurrence prediction models; Table S6: Multivariate Cox proportional hazards analysis for disease-free survival; Figure S1: Internal validation and error convergence of SVM and RF models; Figure S2: Detailed visualization of Al-based recurrence prediction results for individual test cases.
