# Supplementary material for: Personalized Prediction of Postoperative Recurrence in Lung Squamous Cell Carcinoma: Integrating AI-Based Nuclear Morphometry and Clinical Data
Source: J Pers Med. 2026 Apr 6;16(4):205. doi: 10.3390/jpm16040205 (PMC13117173; doi:10.3390/jpm16040205)
Supplement: Supplementary file 1 [file jpm-16-00205-s001.zip › jpm-4189816-supplementary.pdf]

(A) RF(OOB error)

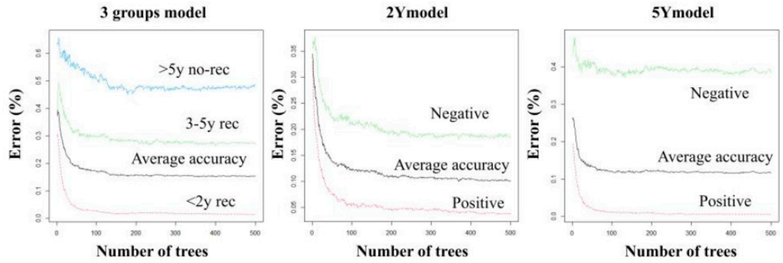

(B) SVM(5-fold cross-validation)

	ROI base						Case base
	model type	5-fold cross validation performance					model type
3 groups model	78.3	74.6	79.0	80.1	80.4	81.7	99.1
2Y model	73.1	79.3	80.1	77.1	79.5	79.7	91.7
5Y model	80.4	79.5	84.4	78.7	82.5	80.5	83.7

Supplementary Figure S1. Internal validation and error convergence of SVM and Rf models. (A) Random forest (RF) out-of-bag (OOB) error curves according to the number of trees for the three-group, 2-year (2Y), and 5-year (5Y) models in the training cohort. (B) Support vector machine (SVM) performance evaluated by 5-fold cross-validation using ROI-level data for the three-group, 2Y, and 5Y models.

								3 group model						2Y model		5Y mode						
								SVM			RF			SVM	RF	SVM	RF					
	Case	Recurrent Y	Folow Y	Rec	Alive/ Death	Stage	Category	<2Y	3-5Y	ΣP※	<2Y	3-5Y	ΣP※	<2Y	<2Y	3-5Y	3-5Y	AI Score	AI Risk	Stage	Total score	Total Risk
1	161	0.7	0.8	+	D	Stage II A	1	0.949	0.001	0.950	0.665	0.043	0.708	0.839	0.692	0.849	0.681	8	H	2	10	H
2	169	0.8	1.8	+	D	StageIIIA	1	0.623	0.000	0.623	0.648	0.051	0.699	0.660	0.650	0.795	0.643	8	H	3	11	H
3	183	0.5	1.0	+	D	Stage II B	1	0.792	0.108	0.900	0.551	0.130	0.681	0.933	0.652	0.740	0.573	8	H	2	10	H
4	170	0.5	1.3	+	D	StageIIIA	1	0.523	0.106	0.629	0.592	0.073	0.665	0.647	0.634	0.464	0.603	7	H	3	10	H
5	154	0.8	1.1	+	D	StageIIIA	1	0.660	0.203	0.863	0.571	0.083	0.654	0.833	0.663	0.614	0.559	8	H	3	11	H
6	148	1.0	6.0	+	D	Stage I A	1	0.563	0.003	0.565	0.533	0.083	0.617	0.587	0.612	0.467	0.509	7	H	1	8	H
7	157	1.5	3.6	+	D	Stage I A	2	0.599	0.035	0.633	0.573	0.124	0.697	0.759	0.672	0.580	0.646	8	H	1	9	H
8	168	1.7	2.3	+	D	Stage II A	2	0.448	0.249	0.697	0.539	0.078	0.618	0.616	0.600	0.376	0.535	6	H	2	8	H
9	178	1.4	3.8	+	A	StageIIIA	2	0.513	0.058	0.570	0.362	0.113	0.475	0.547	0.508	0.438	0.364	4	M	3	7	H
10	162	1.5	1.6	+	D	Stage II A	2	0.546	0.145	0.691	0.569	0.063	0.633	0.832	0.632	0.581	0.560	8	H	2	10	H
11	137	1.6	2.4	+	D	Stage I A	2	0.344	0.496	0.840	0.483	0.092	0.575	0.576	0.595	0.224	0.463	4	M	1	5	M
12	165	2.0	2.0	+	D	Stage II B	3	0.010	0.939	0.948	0.214	0.421	0.635	0.542	0.620	0.143	0.226	5	M	1	6	M
13	182	2.1	3.4	+	A	Stage I A	3	0.024	0.637	0.660	0.233	0.282	0.515	0.149	0.500	0.011	0.241	4	M	1	5	M
14	143	3.5	4.7	+	D	Stage I B	4	0.075	0.892	0.967	0.162	0.322	0.484	0.806	0.469	0.205	0.183	3	M	1	4	M
15	152	4.1	5.0	+	D	Stage I B	4	0.078	0.671	0.749	0.232	0.394	0.626	0.613	0.650	0.178	0.204	5	H	1	6	M
16	181	3.9	5.9	+	A	StageIIIA	4	0.394	0.502	0.896	0.390	0.311	0.702	0.759	0.673	0.397	0.373	5	H	3	8	H
17	2		2.1	-	D	StageIIIA	5	0.347	0.243	0.590	0.201	0.159	0.360	0.642	0.353	0.261	0.190	2	M	3	5	M
18	7		2.6	-	A	Stage I A	5	0.442	0.009	0.451	0.333	0.145	0.477	0.635	0.475	0.587	0.341	2	L	1	3	M
19	25		3.2	-	A	Stage II A	6	0.110	0.003	0.113	0.248	0.125	0.373	0.380	0.366	0.333	0.245	0	L	2	2	L
20	36		3.5	-	A	Stage I B	6	0.680	0.056	0.736	0.301	0.180	0.480	0.905	0.531	0.635	0.301	5	H	1	6	M
21	43		3.8	-	D	Stage I A	6	0.002	0.993	0.995	0.264	0.261	0.525	0.975	0.543	0.679	0.248	6	H	1	7	H
22	18		4.3	-	A	Stage I A	6	0.354	0.205	0.559	0.296	0.182	0.478	0.683	0.482	0.229	0.289	2	L	1	3	M
23	9		4.4	-	A	StageIIIA	6	0.035	0.026	0.061	0.313	0.135	0.448	0.084	0.417	0.080	0.331	0	L	3	3	M
24	20		4.7	-	A	Stage I A	6	0.057	0.254	0.311	0.367	0.161	0.528	0.265	0.527	0.087	0.362	2	L	1	3	M
25	61		4.9	-	D	Stage I A	6	0.173	0.003	0.176	0.169	0.184	0.353	0.627	0.381	0.063	0.162	1	L	1	2	L
26	62		5.0	-	A	Stage I A	7	0.229	0.075	0.304	0.199	0.174	0.373	0.327	0.351	0.246	0.191	0	L	1	1	L
27	53		5.1	-	A	Stage I A	7	0.029	0.012	0.040	0.151	0.096	0.247	0.241	0.219	0.127	0.146	0	L	1	1	L
28	55		5.1	-	A	Stage I B	7	0.239	0.383	0.622	0.235	0.208	0.443	0.467	0.449	0.143	0.226	1	L	1	2	L
29	77		5.4	-	A	Stage I A	7	0.209	0.316	0.525	0.246	0.092	0.338	0.718	0.334	0.287	0.234	2	L	1	3	M
30	81		5.8	-	D	Stage II A	7	0.264	0.159	0.423	0.267	0.118	0.386	0.498	0.386	0.361	0.267	0	L	2	2	L
31	87		6.2	-	A	Stage I A	7	0.104	0.039	0.142	0.152	0.108	0.260	0.274	0.316	0.191	0.144	0	L	1	1	L
32	11		6.5	-	A	Stage II A	7	0.289	0.165	0.454	0.333	0.145	0.478	0.505	0.470	0.162	0.337	1	L	2	3	M
33	94		6.6	-	A	Stage I A	7	0.007	0.002	0.010	0.183	0.099	0.283	0.022	0.282	0.028	0.206	0	L	1	1	L
34	97		6.8	-	A	StageIIIA	7	0.392	0.124	0.516	0.324	0.068	0.392	0.538	0.416	0.280	0.324	2	L	2	4	M
35	3		7.0	-	A	Stage I B	7	0.159	0.076	0.235	0.108	0.075	0.183	0.066	0.156	0.074	0.113	0	L	1	1	L
36	106		7.5	-	D	Stage I B	7	0.218	0.046	0.264	0.164	0.153	0.318	0.282	0.352	0.187	0.160	0	L	2	2	L
37	92		7.9	-	A	Stage I A	7	0.323	0.115	0.439	0.277	0.126	0.403	0.455	0.374	0.321	0.292	0	L	1	1	L
38	99		8.4	-	D	Stage II A	7	0.056	0.501	0.557	0.223	0.085	0.308	0.354	0.354	0.158	0.219	2	L	2	4	M
39	116		9.0	-	A	Stage I A	7	0.351	0.228	0.579	0.259	0.180	0.439	0.532	0.474	0.240	0.273	2	L	1	3	M
40	82		9.5	-	A	Stage I B	7	0.134	0.010	0.145	0.177	0.127	0.304	0.161	0.289	0.240	0.183	0	L	1	1	L
41	57		9.6	-	A	Stage I B	7	0.005	0.326	0.331	0.120	0.150	0.270	0.244	0.253	0.031	0.125	0	L	1	1	L
42	117		10.7	-	A	Stage I A	7	0.097	0.005	0.102	0.171	0.164	0.334	0.331	0.324	0.098	0.170	0	L	1	1	L
43	95		10.9	-	A	Stage II A	7	0.083	0.090	0.173	0.150	0.063	0.213	0.129	0.209	0.031	0.156	0	L	2	2	L
44	108		9.5	-	A	Stage I A	7	0.007	0.034	0.042	0.119	0.098	0.217	0.152	0.206	0.045	0.124	0	L	1	1	L
45	76		9.4	-	A	Stage I A	7	0.008	0.125	0.133	0.144	0.095	0.239	0.306	0.249	0.010	0.140	0	L	1	1	L
46	123		11.4	-	A	StageIIIA	7	0.269	0.053	0.322	0.195	0.179	0.374	0.539	0.348	0.496	0.221	1	L	3	4	M
47	124	5.0	5.4	+	A	Stage I B	9	0.057	0.037	0.093	0.232	0.117	0.350	0.163	0.355	0.073	0.219	0	L	1	1	L
48	100	6.3	6.3	+	D	Stage I B	9	0.002	0.998	1.000	0.265	0.146	0.410	0.898	0.395	0.289	0.266	3	M	1	4	M
49	127	7.5	9.6	+	D	Stage I B	9	0.169	0.318	0.487	0.225	0.239	0.464	0.414	0.499	0.174	0.212	0	L	1	1	L

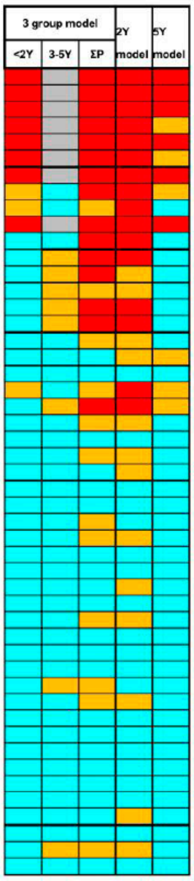

※ΣP : Summation of recurrence likelihood for the <2Y and 3–5Y

strongly suspect recurrence
slight suspicion of recurrence
strong suspicion of no-recurrence
slight suspicion of no-recurrence

	score
SVM/RF both positive (≥0.5)	2
SVM or RF positive	1
SVM/RF both negative (<0.5)	0

AI score/ risk
High risk (6-10)
Middle risk (3-5)
Low risk (1-2)

Total score/risk (Total score = AI score + stage)
High risk (≥7)
Middle risk (3-6)
Low risk (1-2)

Supplemental Figure S2. Detailed visualization of AI-based recurrence prediction results for individual test cases. This heatmap provides a case-level breakdown of the recurrence likelihoods calculated by the six individual AI models (SVM and RF for 2-year, 5-year, and three-category models). The rows represent individual patients in the test cohort, and the columns show the numerical probability scores generated by each algorithm, Color coding corresponds to the risk categories defined in Figure3: red indicates a high likelihood of recurrence (concordant positive), yellow indicates partial positivity, and blue indicates a low likelihood of recurrence (concordant negative). Actual clinical recurrence outcomes and pathologic stages are also displayed to allow for direct comparison with AI-based predictions. *Abbreviations: SVM, support vector machine, RF, random forest*.

Supplemental Table S1. The number of ROIs and nuclei in each category of the analyzed cases.

		Training			Test			Total		
category		Case	ROI	Nuclei	Case	ROI	Nuclei	Case	ROI	Nuclei
1	Rec(+) < 1Y	17	400	357744	6	142	128002	23	542	485746
2	Rec(+) 1~2Y	13	264	201398	5	120	80540	18	384	281938
3	Rec(+) 2~3Y	8	161	114985	2	31	19090	10	192	134075
4	Rec(+) 3~5Y	8	161	146032	3	64	61123	11	225	207155
5	Rec(-) 2~3Y	4	73	45372	2	45	27987	6	118	73359
6	Rec(-) 3~5Y	20	442	351575	7	143	116378	27	585	467953
7	Rec(-) > 5Y	60	1331	1072098	21	460	330972	81	1791	1403070
9	Rec(+) > 5Y	6	109	89735	3	48	36959	9	157	126694
Total		136	2941	2378939	49	1053	801051	185	3994	3179990

Supplemental Table S2. The results of SVM and RF models in the three-group AI model.

(i) SVM model

(A) ROI-based Training set result — Accuracy = 96.7%

		Prediction			
	Group	No recurrence	< 2y recurrence	2-5y recurrence	total
Actual	No recurrence	1305	25	1	1331
	< 2y recurrence	51	613	0	664
	2-5y recurrence	0	0	321	321
	total	1356	638	322	2316

(B) ROI-based Test set result — Accuracy = 78.3%

		Prediction			
	Group	No recurrence	< 2y recurrence	2-5y recurrence	total
Actual	No recurrent	373	47	40	460
	< 2y recurrence	55	187	20	262
	2-5y recurrence	8	7	80	95
	total	436	241	140	817

(C) Case-based Training set result — Accuracy = 99.1%

		Prediction			
	Group	No recurrence	< 2y recurrence	2-5y recurrence	total
Actual	No recurrent	60	0	0	60
	< 2y recurrence	1	29	0	30
	2-5y recurrence	0	0	16	16
	total	61	29	16	106

(D) Case-based Test set result — Accuracy = 94.6%

		Prediction			
	Group	No recurrence	< 2y recurrence	2-5y recurrence	total
Actual	No recurrent	19	0	2	21
	< 2y recurrence	0	11	0	11
	2-5y recurrence	0	0	5	5
	total	19	11	7	37

ROI, region of interest; SVM, support vector machine; RF, random forest; y, years

(ii) RF model

(E) ROI-based Training set result — Accuracy = 100.0%

		Prediction			
	Group	No recurrence	< 2y recurrence	2-5y recurrence	Total
Actual	No recurrence	1331	0	0	1331
	< 2y recurrence	0	664	0	664
	2-5y recurrence	0	0	321	321
	total	1331	664	321	2316

(F) ROI-based Test set result — Accuracy = 79.3%

		Prediction			
	Group	No recurrence	< 2y recurrence	2-5y recurrence	total
Actual	No recurrent	443	17	0	460
	< 2y recurrence	84	178	0	262
	2-5y recurrence	54	14	27	95
	total	581	209	27	817

(G) Case-based Training set result — Accuracy = 100.0%

		Prediction			
	Group	No recurrence	< 2y recurrence	2-5y recurrence	total
Actual	No recurrent	60	0	0	60
	< 2y recurrence	0	30	0	30
	2-5y recurrence	0	0	16	16
	total	60	30	16	106

(H) Case-based Test set result — Accuracy = 91.9%

		Prediction			
	Group	No recurrence	< 2y recurrence	2-5y recurrence	total
Actual	No recurrent	21	0	0	21
	< 2y recurrence	0	11	0	11
	2-5y recurrence	2	1	2	5
	total	23	12	2	37

ROI, region of interest; SVM, support vector machine; RF, random forest; y, years

Supplemental Table S3. The results of SVM and RF models in the 2-year recurrence model. (only Test set result)

(i) SVM model

(A) ROI-based Test set result — Accuracy = 73.1%

Group	No recurrence	< 2 y recurrence	Total
No recurrence	324	136	460
< 2 y recurrence	84	273	357
total	408	409	817

(B) Case-based Test set result — Accuracy = 91.7%

Group	No recurrence	< 2 y recurrence	Total
No recurrence	18	2	20
< 2 y recurrence	1	15	16
total	19	17	36

(ii) RF model

(C) ROI-based Test set result — Accuracy = 80.4%

Group	No recurrence	< 2 y recurrence	Total
No recurrence	388	72	460
< 2 y recurrence	88	269	357
total	476	341	817

(D) Case-based Test set result — Accuracy = 97.2%

Group	No recurrence	< 2 y recurrence	Total
No recurrence	20	0	20
< 2 y recurrence	1	15	16
total	21	15	36

ROI, region of interest; SVM, support vector machine; RF, random forest; y, years

Supplemental Table S4. The results of SVM and RF models in the 5-year recurrence model. (only Test set result)

(i) SVM model

(A) ROI-based Test set result — Accuracy = 80.4%

Group	No recurrence	< 5 y recurrence	Total
No recurrence	690	101	791
< 5 y recurrence	105	157	262
total	795	258	1053

(B) Case-based Test set result — Accuracy = 83.7%

Group	No recurrence	< 5 y recurrence	Total
No recurrence	35	3	38
< 5 y recurrence	5	6	11
total	40	9	49

(ii) RF model

(C) ROI-based Test set result — Accuracy = 88.6%

Group	No recurrence	< 5 y recurrence	Total
No recurrence	777	14	791
< 5 y recurrence	106	156	262
total	883	170	1053

(D) Case-based Test set result — Accuracy = 95.9%

Group	No recurrence	< 5 y recurrence	Total
No recurrence	38	0	38
< 5 y recurrence	2	9	11
total	40	9	49

ROI, region of interest; SVM, support vector machine; RF, random forest; y, years

Supplemental Table S5. Relative importance of nuclear morphological and textural features in AI-based recurrence prediction models.

		SVM		RF	
		2Ymodel	5Ymodel	2Ymodel	5Ymodel
Nuclear shape related features	Area	2.9%	4.9%	4.6%	3.4%
	Compactness	0.5%	1.0%	4.8%	5.1%
	Eccentricity	0.8%	2.4%	2.3%	2.4%
	Extent	2.0%	1.3%	2.3%	2.6%
	FormFactor	3.5%	6.3%	2.7%	2.4%
	MajorAxisLength	2.2%	1.7%	3.3%	3.0%
	MaxFeretDiameter	1.4%	2.2%	2.8%	3.2%
	MaximumRadius	9.0%	10.3%	2.5%	2.9%
	MeanRadius	3.6%	3.8%	2.1%	2.4%
	MedianRadius	3.9%	5.5%	3.4%	3.0%
	MinFeretDiameter	2.0%	6.8%	3.5%	3.5%
	MinorAxisLength	3.9%	3.3%	3.0%	3.1%
	Orientation	41.8%	26.1%	2.2%	1.9%
	Perimeter	1.3%	0.7%	2.6%	2.8%
	Solidity	1.5%	2.2%	2.8%	2.4%
	FracAtD_ratio	4.0%	2.0%	4.3%	3.7%
	MeanFrac_ratio	1.6%	1.6%	3.2%	3.3%
	Total	85.8%	82.0%	52.4%	51.1%
Intranuclear texture related features	AngularSecondMoment_max	0.6%	0.8%	3.2%	4.4%
	Contrast_max	1.1%	1.0%	6.5%	6.2%
	Correlation_max	0.4%	0.8%	5.3%	4.6%
	DifferenceEntropy_max	1.3%	1.7%	2.9%	2.5%
	DifferenceVariance_max	0.6%	0.7%	5.2%	4.4%
	Entropy_max	1.3%	2.9%	2.9%	3.3%
	InfoMeas1_max	0.5%	0.9%	3.3%	3.2%
	InfoMeas2_max	1.4%	2.2%	3.3%	3.2%
	InverseDifferenceMoment_max	1.4%	0.6%	2.7%	2.7%
	SumAverage_max	2.1%	1.3%	2.8%	3.0%
	SumEntropy_max	1.6%	1.1%	3.4%	4.2%
	SumVariance_max	1.3%	3.2%	3.2%	3.3%
	Variance_max	0.6%	0.9%	3.0%	4.0%
	Total	14.2%	18.0%	47.6%	48.9%
		100.0%	100.0%	100.0%	100.0%

Supplemental Table S6. Multivariate Cox proportional hazards analysis for disease-free survival

Variables	Hazard ratio	95%CI	p
Pathological stage	1.65	1.18 - 2.31	0.004
Differentiation	0.79	0.53 - 1.18	0.258
Tumor size	1.11	0.96 - 1.28	0.164
Pleural invasion	1.29	0.94 - 1.76	0.110
Lymphoid invasion	1.35	0.77 - 2.38	0.297
